# Altered sensitivity to social gaze in the *FMR1* premutation and pragmatic language competence

**DOI:** 10.1186/s11689-017-9211-z

**Published:** 2017-08-24

**Authors:** Jessica Klusek, Joseph Schmidt, Amanda J. Fairchild, Anna Porter, Jane E. Roberts

**Affiliations:** 1 Department of Communication Sciences and Disorders, University of South Carolina, Keenan Building 1229 Marion Street, Suite 300, Columbia, SC 29208 USA; 2 Department of Psychology, College of Sciences, University of Central Florida, 4111 Pictor Lane, Orlando, FL 32816 USA; 3 Department of Psychology, University of South Carolina, 1512 Pendleton Street, Columbia, SC 29208 USA

**Keywords:** Fragile X carriers, Eye contact, Social communication, Direct gaze, Social cognition

## Abstract

**Background:**

The *FMR1* premutation affects 1:291 women and is associated with a range of cognitive, affective, and physical health complications, including deficits in pragmatic language (i.e., social language). This study investigated attention to eye gaze as a fundamental social-cognitive skill that may be impaired in the *FMR1* premutation and could underlie pragmatic deficits. Given the high prevalence of the *FMR1* premutation, efforts to define its phenotype and mechanistic underpinnings have significant public health implications.

**Methods:**

Thirty-five women with the *FMR1* premutation and 20 control women completed an eye-tracking paradigm that recorded time spent dwelling within the eye region in response to a face displaying either direct or averted gaze. Pragmatic language ability was coded from a conversational sample using the Pragmatic Rating Scale.

**Results:**

Women with the *FMR1* premutation failed to show attentional preference to direct gaze and spent more time dwelling on the averted eyes relative to controls. While dwelling on the eyes was associated with better pragmatic language performance in controls, these variables were unrelated in the women with the *FMR1* premutation.

**Conclusions:**

Altered sensitivity to social gaze, characterized by increased salience of averted gaze, was observed among women with the *FMR1* premutation. Furthermore, women with the *FMR1* premutation were unable to capitalize on information conveyed through the eyes to enhance social-communicative engagement, which differed from patterns seen in controls. These findings contribute to the growing characterization of social and communication phenotypes associated with the *FMR1* premutation.

## Background

The *Fragile X Mental Retardation-1* (*FMR1*) premutation is a prevalent genetic alteration affecting 1 in 291 women [1]. The premutation occurs when the CGG element on the 5′ untranslated region of the *FMR1* expands within the range of 55–200 repeats [2]. The expanded CGG sequence shows generational instability and women “carriers” of the *FMR1* premutation are at risk of passing the mutation to their offspring, which causes fragile X syndrome when the expansion exceeds 200 repeats [3].

Besides conferring familial risk, the *FMR1* premutation is associated with its own clinical consequences, which include increased risk for mental health disorders [4], mild cognitive deficits [5], and physical health complications such as fragile X-associated premature ovarian insufficiency and fragile X-associated tremor ataxia syndrome (FXTAS; [6, 7]). Much existing research on the *FMR1* premutation has focused on men, who show social-cognitive difficulties such as decreased ability to “read” mental and emotional states from the eyes [8]. Atypical brain and autonomic responses to social stimuli, such as reduced conductance response when greeting an unfamiliar person and diminished amygdala activation when viewing fearful faces, are also seen in males with the premutation [9, 10]. Less research has focused on females, but emerging work suggests a similar profile of social-cognitive weaknesses. For instance, risk for autism is increased among females with the premutation, with about 5% meeting criteria for autism spectrum disorder [11]. Women with the *FMR1* premutation also exhibit increased personality features consistent with the broad autism phenotype [12] and decreased sensitivity to biological motion [13]. Yet, the phenotypic signature of the *FMR1* premutation is not fully understood, and few investigations have focused on language features in this group.

Difficulty with pragmatic language (i.e., social language) is a recently documented feature of the *FMR1* premutation that is prime for further study, given its clinical relevance. As a group, women with the *FMR1* premutation commit more pragmatic language violations during conversation than do control women, which includes features such as over-talkativeness and introducing inappropriate topics [12]. At the family level, these features are clinically significant; pragmatic language difficulties among mothers with the *FMR1* premutation are associated with poor language outcomes for their children with fragile X syndrome [14]. Additionally, these pragmatic language features may have negative consequences at the individual level. Pragmatic impairments are associated with lower quality friendships [15], social rejection [16], emotional difficulties [17], and feelings of loneliness [18] in other clinical groups, although individual effects have not yet been studied in the premutation.

While impaired use of eye gaze to infer mental/emotional states has been documented among men with the premutation (i.e., [8]), social-cognitive abilities involving the eye gaze have not been investigated among women with the *FMR1* premutation. The present study sought to address this gap in the literature, with a focus on differential sensitivity to direct and averted gaze. The eyes represent a powerful modality for gleaning information about the social world, lending clues into the thoughts, beliefs, desires, and emotional state of others [19]. Sensitivity to the direction of eye gaze is hypothesized to represent a biologically prepared skill, as very young infants exhibit enhanced neural processing in response to direct gaze and are able to distinguish direct from averted gaze [20, 21]. This innate sensitivity to eye gaze is crucial to the development of theory of mind, or the ability to attribute mental states to others, and is thought to provide the foundation for the later development of social and communication skills [20, 22]. Human preference for direct gaze over averted gaze is well documented, with more efficient detection of direct gaze during visual search tasks [23, 24] and longer disengagement latencies in response to direct gaze [25]. The increased saliency of direct gaze has evolutionary significance, as direct gaze represents a primary communicative channel by which humans exchange social-cognitive information, facilitating adaptive social engagement [26]. Consistent with this notion, research shows that direct gaze facilitates the retrieval of social-cognitive knowledge, with enhanced recognition of emotions, faces, speech, and gender observed under direct gaze conditions relative to averted gaze [27–33]. In sum, the eyes, and the direction of eye gaze in particular, represent a valuable source of information needed for social approach and engagement.

A number of empirical studies support a role of social gaze in language acquisition [34–37]. According to the social-pragmatic theory, social-cognitive skills, such as the ability to “read the mind from the eyes,” are fundamental to language development because they allow communicative partners to establish a shared context that can be used to infer the meaning behind words and utterances [38, 39]. Eye gaze in particular represents a medium through which communication partners exchange critical information about intentions, moods, and emotions [40, 41]. During conversation, conversational partners spend a great deal of time attending closely to others’ eye gaze patterns [42–44]. Eye gaze provides information on speaker intent and can be useful in resolving conversational ambiguities [45] and in detecting deception [46]. Information on the conversational partner’s interest and understanding is also conveyed through gaze, allowing the speaker to adopt their message to enhance the listener’s engagement and comprehension [43, 47]. Eye gaze is also used to establish listener/speaker roles so that disruptive turn-taking, such as interruptions and overlapping speech, are kept at a minimum [48, 49]. Thus, suboptimal use of gaze signals can lead to a number of maladaptive conversational strategies. This study tested the hypothesis that aberrant attention to social gaze contributes to pragmatic deficits in the *FMR1* premutation.

### The present study

This study employed an eye-tracking paradigm to record the time spent dwelling on the eyes in response to an animated face displaying either direct or averted gaze. The study’s first objective was to determine whether attention to the eyes in response to direct gaze and averted gaze, reflecting perceived salience, differed across women with the *FMR1* premutation and control women. The second aim was to determine whether attention to the eyes under direct and averted gaze conditions was associated with pragmatic language variation in these groups. The *FMR1* premutation is a highly prevalent genetic condition, making efforts to define its phenotype and associated mechanisms a public health imperative. This work is also important because of its implications for understanding the underpinnings of language behaviors relevant to both atypical and typical populations as they present during adulthood—an understudied and underserved developmental period. The following research questions were tested:

1. Does attention to the eyes in response to direct and averted gaze differ in women with the *FMR1* premutation contrasted to control women? *It was hypothesized that women with the FMR1 premutation would spend less time dwelling on the eyes than control women, reflecting social-cognitive weaknesses in this group. It was hypothesized that both groups would dwell on the eyes longer in the direct gaze condition than in the averted gaze condition, consistent with evidence supporting preferential attention to direct gaze*.
2. Is attention to the eyes in response to direct and averted gaze associated with pragmatic language variation in women with the *FMR1* premutation contrasted to control women? *Consistent with the notion that eye gaze is a necessary source of information for social engagement, it was hypothesized that dwelling on the eyes in response to direct and averted gaze would be associated with increased pragmatic language competence in both groups*.

## Methods

### Participants

Thirty-five women with the *FMR1* premutation and 20 control women participated. Participants were drawn from a larger study of social-language in women with the *FMR1* premutation that has been previously described﻿ (Klusek et al., [50]). All participants were native speakers of English, were mothers, had normal or corrected-to-normal visual acuity, and did not have an intellectual disability as defined by an IQ composite >80 on the Kaufman Brief Intelligence Test-II [51]. Women with the *FMR1* premutation were recruited through their children who were participating in developmental studies of children with the fragile X full mutation or the premutation (PI’s: Abbeduto, Roberts) or were recruited through the local community through word of mouth targeting mothers of children with fragile X or the premutation. Recruitment was based in the Eastern and Midwestern regions of the United States of America through advertisements on social media, parent support networks, and with the assistance of the Research Participant Registry Core of the Carolina Institute for Developmental Disabilities. The *FMR1* premutation was confirmed via genetic testing and defined by an allele ranging from 55 to 200 CGG repeats on *FMR1*. None of the women with the premutation had been diagnosed with FXTAS, per self-report, and functional symptoms of tremor were screened out using the Tremor Disability Questionnaire [52]. Control women had no known family history of fragile X and were mothers of typically developing children (i.e., children who had not been diagnosed or treated for any type of developmental delay or disorder, per participant report). Although it was beyond the scope of the present study to conduct genotyping on controls, 85% of the control sample completed blood tests to rule out the *FMR1* premutation through dual enrollment in a related pilot study. Women in the control group were excluded from the study if their child scored above the cut-off for autism spectrum disorder on the Social Communication Questionnaire (*n* = 1) [53]. The groups did not differ significantly on age, IQ, race, or education level; see Table 1.

**Table 1:** Group characteristics

Variable	Group		
	Women with the *FMR1* premutation (*n* = 35)	Control women (*n* = 20)	Test of group differences (*p* value)
Age in years			
M (SD)	45.81 (8.34)	43.10 (9.70)	.291
Range	26.55–59.96	32.50–65.23	
IQ^1^			
M (SD)	103.46 (11.80)	106.74 (9.99)	.330
Range	81.00–126.00	91.00–135.00	
Race (%)			
Caucasian	87	85	.323
African American	3	10	
Other	10	5	
Highest education level (%)			
High school or lower	17	30	.347
Bachelor’s degree	55	35	
Master’s degree	24	25	
Professional degree	4	10	

### Procedure

Assessments were conducted at a university laboratory setting, within the context of a larger research protocol that lasted approximately 3 h. Participants were compensated $50.00 for their time. Informed consent was obtained, and all procedures were approved by the Institutional Review Board of the University of South Carolina.

### Eye gaze paradigm

Participants were seated in a quiet, well-lit room. Eye movements were recorded using a desktop mounted Eyelink 1000 Plus (SR Research Ltd., Ontario, Canada) operating in remote mode, which provides compensation for head movements. Eye movements were sampled at 500 Hz, and tracking was monocular. The experiment was programed and run using the Experiment Builder software (version 1.10 SR Research Ltd., Ontario, Canada). Saccades and fixations were parsed based on the online velocity and acceleration of the eye using default parameters built into the EyeLink 1000 Plus software. Stimuli were presented on a BEN-Q 2420T monitor, displayed at the resolution of 1366 × 768 pixels and a 60 Hz refresh rate. The size of the screen was 530 × 300 mm, viewed from a distance of 950 mm (allowing for a maximum visual angle of 31**°** in the horizontal dimension and 18**°** in the vertical dimension). An initial 5-point calibration and validation were performed at the start of the procedure. Calibrations were not accepted until average, and maximum errors were less than .5° and 1.00°, respectively. Each trial was preceded by a centrally presented single point drift check that recorded any drift in the eye data before the trial began. Recalibrations were conducted as needed to prevent drift.

**Fig. 1:**
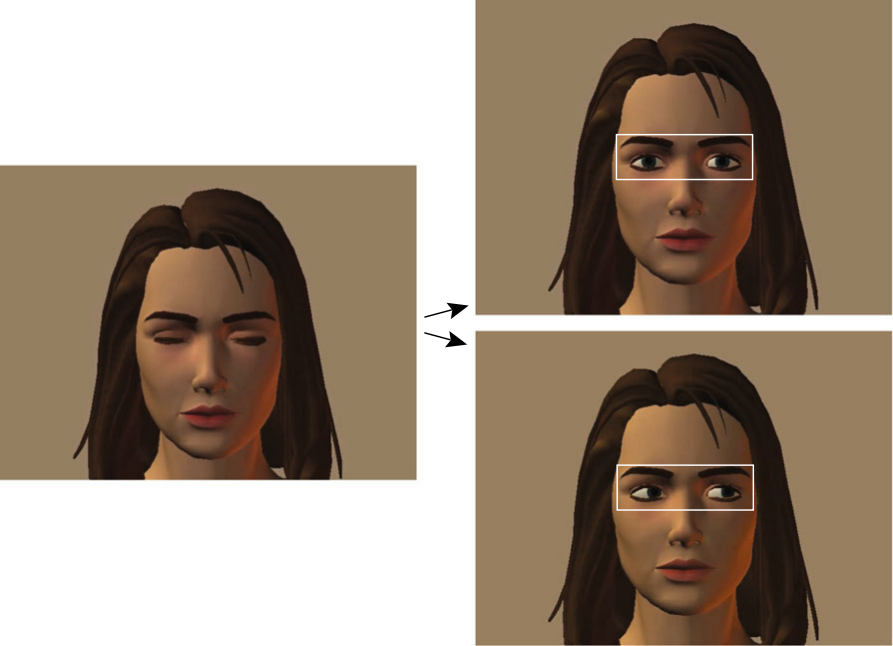
Example stimuli from the eye gaze paradigm. Note*.* Example stimuli from the eye gaze paradigm are presented. Trials begin with the eyes closed (*leftmost image*). After the eyes open, the face displays either direct gaze (*top right*) or averted gaze (*bottom right*). The eye area of interest is marked with a white rectangle

Stimuli and procedures were adapted from Weiser et al. [54]. The eye gaze paradigm consisted of a series of short video sequences where a computer-generated female face (which subtended 7° in the horizontal and 14° in the vertical dimension) started with her eyes closed and then opened her eyes to display either direct or averted gaze (see Fig. 1, for example, stimuli). At the start of the task, participants were instructed: “You are going to see a series of faces on the screen. The faces will have their eyes closed. I want you to look within the eye region of the face until the eyes open. After the eyes open, you can look anywhere you want on the screen.” Each trial initially depicted the face with the eyes closed for a minimum of 1200 ms. Once the participant was looking within the eye region for an additional 300 consecutive milliseconds (resulting in a minimum eyes-closed duration of 1500 ms), the eyes opened. After the eyes opened, the face remained in the direct or averted gaze position for 6000 ms. Individual trials were separated by an inter-trial-interval ranging from 300 to 400 ms, which consisted of a blank screen. The female faces were displayed on a gray background and showed a neutral facial expression. Stimuli were presented so that the eye region area of interest (defined by a rectangle drawn around the eyes which subtended 8° in the horizontal and 3° in the vertical dimension) was centered on the screen. Overall, 16 different female characters were used, each displaying three possible gaze conditions: direct, averted to the left, or averted to the right. Stimuli for each condition (direct, averted) were displayed at random until 16 trials of each condition had been shown. In the averted condition, the direction of eye gaze (right, left) was randomly selected. Only data from the first 16 trials of each condition were analyzed, yielding a total of 32 trials. Dataviewer (version 2.41 SR Research, Ontario, Canada) was used to extract the variables of interest. Dataviewer loads all of the online-parsed fixations, saccades, blinks, and samples. Eye movement data is then mapped to the experiment interest areas and images for more detailed analyses. Eye movement data were analyzed in terms of the percent dwell time on the eye interest area across all trials within each condition. On each trial, the cumulative fixation duration (i.e., excluding time spent making saccades and blinks) falling within the eye interest area was divided by the cumulative fixation duration across all fixations in the trial. This proportion was then multiplied by 100 to indicate the percentage of total fixation time spent on the eye region.

### Pragmatic language assessment

Pragmatic language ability was rated from a 20-min conversational sample in which participants conversed with an examiner about their “life history.” Standard probe questions were used to elicit conversation on neutral, shared experiences, such as “What activities did you enjoy most as a child” and “Did you participate in extra-curricular activities in high school?” In addition to administering the standard probe questions, examiners commented, offered information, and asked follow-up questions to ensure ample opportunities for conversational exchange. Pragmatic ability was coded from the videotaped conversational samples using the modified version of the Pragmatic Rating Scale [55], which has been described previously as a tool for capturing subclinical pragmatic difficulties in relatives of children with developmental disorders (see [12, 56]). The scale consists of 26 items that are coded based on operational definitions capturing the frequency and severity of each potential violation, where generally a “0” denotes the violation is absent, a “1,” mild or questionable, and a “2,” present and striking. Example items include “fails to provide background information,” “pedantic word choice,” and “unusual intonation.” A total score denoting the severity of pragmatic language difficulties is computed by tallying the items. Two trained independent raters coded each sample and disagreements were resolved through discussion. Consensus codes were used in the analysis. The second rater was blind to the group membership of all participants, whereas the first rater (JK) was unable to remain blinded due to her role in participant assessment. Inter-rater reliability of the total score prior to consensus was ICC (3, 2) = 0.73 which is considered “good” [57].

### Data analysis

Analyses were conducted in SAS 9.4 [58]. The data distribution was first examined, and all variables were normally distributed. To determine group differences in the percent dwell time on the eyes across direct and averted gaze conditions (Research Question 1), a mixed effects linear model was fit to test for group differences in the percent of time dwelling within the eye region of the face across conditions. Condition (averted or direct gaze) was specified as a random effect, nested within participant. Group, condition, and their interaction were included as predictors. An unstructured covariance matrix was specified. Analyses were conducted to determine whether the percent dwell time on the eyes across conditions predicted pragmatic language ability across the groups (Research Question 2). General linear models were conducted to test dwell time, group, and their interaction as predictors of the pragmatic language total score. Separate models were run for the averted and direct gaze conditions, and the Benjamini-Hochberg correction procedure [59] was used to control for false discovery by adjusting critical values for the model *F* test. Interaction contrasts were calculated to determine the effect of dwell time on pragmatic language competency at each level of group. Partial eta squared (*η* ^2^_p_) effect sizes were computed. In general, values of *η* ^2^_p_ at 0.01, 0.06, and 0.14 are considered “small,” “medium,” and “large,” respectively [60].

## Results

### Descriptive statistics

Descriptive statistics for the predictor and outcome variables are reported in Table 2. The groups differed significantly on the pragmatic language total score, *t* (39.62) = 3.57, *p* = .001, with elevated pragmatic language violations in the women with the *FMR1* premutation relative to controls.

**Table 2:** Descriptive statistics

Variable	Group	
	*FMR1* premutation	Control
Pragmatic language total score *M* (SD), range	9.35 (3.71), 2.00–15.00	5.6 (3.74), 1.00–16.00
Percent of time dwelling on eyes (averted gaze) M (SD), range	0.62 (0.18), 0.23–0.95	0.57 (0.21), 0.30–0.89
Percent of time dwelling on eyes (direct gaze) M (SD), range	0.62 (0.18), 0.25–0.92	0.62 (0.24), 0.27–0.96

### Group differences in time dwelling on the eyes

**Fig. 2:**
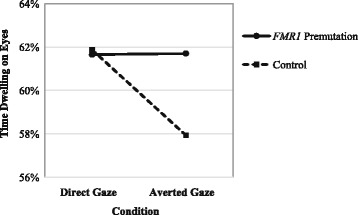
Time dwelling on the eyes across groups and conditions

The mixed effects model showed a significant main effect for condition (*F* [1, 53] = 5.22, *p* = .026) and a non-significant main effect for group (*F* [1, 53] = 0.11 *p* = .744). A significant group-by-condition interaction was detected, *F* (1, 53) = 5.51, *p* = .023. The control women spent less time dwelling on the eyes in the averted gaze condition relative to relative to direct gaze, whereas the dwell times of the women with the *FMR1* premutation did not vary by condition. Relative to the controls, the women with the *FMR1* premutation spent significantly more time dwelling on the eyes in the averted condition, whereas groups spent a similar amount of time dwelling on the eyes in the direct gaze condition (see Fig. 2).

### Association between time dwelling on the eyes and pragmatic language ability

#### Direct gaze model

**Fig. 3:**
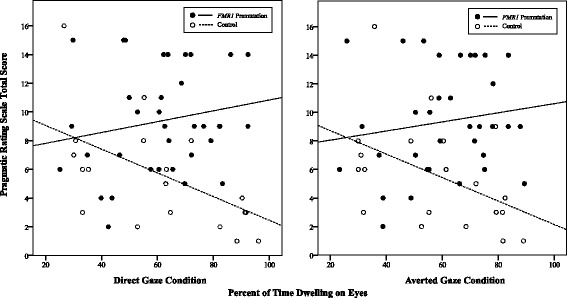
Association between time dwelling on the eyes during the direct gaze condition and pragmatic language ability. Note. A higher total score on the Pragmatic Rating Scale denotes increased pragmatic language difficulties

The combined influence of group, time dwelling on the eyes in the direct gaze condition, and their interaction accounted for significant variance in pragmatic language ability, *F* (3, 50) = 7.11, *p* < .001, *R* ^2^ = .30. The group-by-condition interaction term was significant (*p* = .016), with a *η* ^2^_p_ of 0.11 consistent with a medium-to-large effect (Cohen, 1969). Interaction contrasts indicated that the effect of dwell time in the direct gaze condition on pragmatic language ability differed by group; among the control women, increased dwell time on the eyes was significantly associated with better pragmatic language skills (*F* [1, 53] = 6.04, *p* = .018, *η* ^2^_p_ = .11 [90% CI = .01–.24]), whereas the time dwelling on the eyes was not associated with pragmatic ability in the *FMR1* premutation (*F* [1, 53] = 1.19, *p* = .280, *η* ^2^_p_ = .02 [90% CI = 0–.12]), see Fig. 3.

#### Averted gaze model

Findings for the averted gaze model were similar, with the combined influence of group, time dwelling on the eyes in the averted condition, and their interaction accounting for significant variance in pragmatic language ability, *F* (3, 50) = 6.18, *p* = .001, *R* ^2^ = .27. A significant group-by-condition interaction was detected (*p* = .039), with an *η* ^2^_p_ of 0.08 consistent with a medium effect (Cohen, 1969). Similar to the direct gaze model, interaction contrasts indicated that the influence of dwell time on pragmatics differed by group membership; increased dwell time in the averted condition was significantly associated with better pragmatic language skills in controls (*F* [1, 53] = 4.20, *p* = .046, *η* ^2^_p_ = .08 [90% CI = 0–.20]), whereas the time dwelling on the eyes was not significantly associated with pragmatic ability among the women with the *FMR1* premutation (*F* [1, 53] = 0.79, *p* = .378, *η* ^2^_p_ = .02 [90% CI = 0–.11]). For both general linear regression models, two observations exceeded recommended cut-off criteria for influence for Cook’s *D* (i.e., Di > 4/n-k-1; [61]); however, inference across solutions including and excluding the observations were identical and thus only the solution associated with complete data is presented here for parsimony. Regression coefficients are presented in Table 3.

**Table 3:** Regression coefficients depicting dwelling on the eyes as a predictor of pragmatic language ability

Effect	B	SE	*t*	*p*	*η* ^2^_p_ (90% CI)	*R* ^2^
Coefficients: direct gaze model						
Intercept	10.72	2.23	4.81	<.001*		.30
Group^a^	−3.66	3.12	−1.17	.247	.02 (0–.13)	
Dwell time (direct)	−8.27	3.37	−.246	.018*	.01 (0–.11)	
Group × dwell time	12.05	4.83	2.50	.016*	.09 (.01–.24)	
Coefficients: averted gaze model						
Intercept	10.36	2.46	4.21	<.001*		.27
Group^a^	−2.94	3.34	−0.88	.383	.02 (0–.11)	
Dwell time (averted)	−8.21	4.01	−2.05	.045*	.02 (0–.11)	
Group × dwell time	11.40	5.38	2.12	.039*	.08 (0–.21)	

^a^The control group was set as the reference category

*p < .05

## Discussion

The *FMR1* premutation is a prevalent genetic mutation associated with a range of cognitive, affective, and physical health complications including pragmatic language deficits that are of clinical significance. The present study represents the first investigation of attention to eye gaze in women with the *FMR1* premutation, with the hypothesis that this fundamental social-cognitive skill may be impaired in this group and may underlie pragmatic language features. Findings showed increased salience of averted gaze among the women with the *FMR1* premutation relative to control women. Additionally, group-specific patterns were detected where greater attention to eye gaze was associated with enhanced pragmatic language competence in control women, but not in women with the *FMR1* premutation. The decoupling of gaze sensitivity and pragmatic language ability in the *FMR1* premutation provides preliminary evidence that the development of social communication skills differs between carriers of the *FMR1* premutation and controls. This study informs the interface between component social-cognitive and language features of the *FMR1* premutation phenotype and highlights future avenues of research aimed at identifying causal mechanisms.

### Increased salience of averted gaze in the *FMR1* premutation

Study controls dwelled longer on the eyes during the direct gaze condition compared to averted gaze, which is consistent with evidence supporting a human preference for direct gaze [23–25]. Our results showed that women with the *FMR1* premutation failed to demonstrate this preferential attention to direct gaze, suggesting that the perceived significance of eye gaze may be aberrant in this group. Notably, the atypical attentional patterns in the premutation group appear to be driven by increased saliency of averted gaze rather than diminished saliency of direct gaze, as time spent dwelling on the eyes was increased relative to controls only in response to the averted condition. Why, then, did the women with the *FMR1* premutation dwell longer on the eyes only in response to averted gaze? The eyes convey significant social information and increased attention to the eyes may be interpreted as an effort to compensate for inefficient processing of social input. Averted gaze, which has more ambiguous meaning than direct gaze, may have required greater effort to process than direct gaze (and hence was associated with longer dwell times). Another possibility is that the women with the *FMR1* premutation simply failed to recognize direct gaze as having increase saliency relative to averted gaze, which would suggest a disruption in innate, biologically-driven recognition of core social cues. Atypical processing of other fundamental social signals has been documented in women with the *FMR1* premutation, such as reduced sensitivity to biological motion (e.g., [13]). This interpretation is also consistent with neuroimaging studies of the *FMR1* premutation documenting reduced activation of the amygdala and superior temporal sulcus [9, 10], which are brain areas implicated in gaze processing and social cognition [62].

It seems most likely that the looking behavior of the women in this study reflected social-cognitive processes, given the large body of research supports the fundamental role of social gaze in the development and exchange of social-cognitive knowledge [20–22, 26–33]. However, we cannot be sure what underlying processes motivated the looking behavior of the women, and it is possible that other factors influenced attention to the eyes. One alternative explanation is that elevated social anxiety drove the attentional vigilance towards averted gaze, as elevated rates of social anxiety have been documented among women with the *FMR1* premutation [63, 64]. Social anxiety is a condition characterized by fear of negative social evaluation and attentional bias for social-evaluative threat cues, and one function of averted gaze is to convey negative relational evaluation and social exclusion [65, 66]. A prior study employing these stimuli in a similar eye-tracking paradigm found that female college students with high levels of social anxiety showed higher mean fixation duration on the eyes than those with low social anxiety, although group differences did not reach statistical significance [54]. It is also possible that executive deficits contributed to the atypical dwelling patterns of the women with the *FMR1* premutation. Women with the *FMR1* premutation show subtle deficits across a range of executive processes [67–69], and executive control would be required to shift attention from the eyes to other parts of the face. Follow-up investigations are needed to clarify the underlying processes responsible for altered sensitivity to social gaze in the *FMR1* premutation.

### Relationship between eye gaze sensitivity and pragmatic language across groups

Contrary to hypotheses, attention to eye gaze was not associated with pragmatic language ability in women with the *FMR1* premutation. Yet, these variables were linked among study controls; control women who attended more carefully to the eyes showed enhanced pragmatic language competence. This finding in controls is consistent with social-pragmatic theory and a large body of research showing that the eyes convey essential social-cognitive information that facilitates social-communicative engagement [38, 39]. Thus, the lack of relationship between gaze processing and pragmatics in the *FMR1* premutation suggests that attending to the eyes did not translate to enhanced registration of social cognitive information in this group. A critical question remains: if attention to social gaze did not correlate with pragmatic language ability in the *FMR1* premutation, what does? Better understanding of the factors associated with pragmatic language difficulties is critical to the development of family-centered prevention/intervention programs that can best support the complex needs of families affected by fragile X-associated conditions.

### Implications for theory, research, and practice

Findings showed a relationship between attention to eye gaze and social-communicative ability in control women that was of a medium-to-large effect. In some respects, it is surprising that the time spent dwelling on the eyes of an animated face in a controlled experimental context was strongly associated with pragmatic performance sampled from an independent, semi-naturalistic communicative interaction. The strength of the association between these behaviors, despite the disparate contexts from which they were measured, underscores the importance of the eyes in navigating social interactions. Although a wealth of empirical studies have established sensitivity to eye gaze as foundational skill for social-communicative engagement, the majority of this work has focused on early development and relatively little is known about the functions and use of eye gaze in adult populations. This study sheds light on social-cognitive correlates of communicative behavior in adults and suggests that the application of social-pragmatic theory is not limited to childhood. While this study examined subtle pragmatic language variation in non-disordered individuals, findings may also have relevance for disordered populations, where the prevalence of adults with social-communication impairments, such as those with autism spectrum disorder, is increasing (e.g., [70]). The importance of understanding the nature and basis of pragmatic language impairment is underscored by the recent addition of social communication disorder as its own diagnostic category in the Diagnostic and Statistical Manual of Mental Health Disorders, which is used by clinicians, researchers, and public health officials to classify mental health disorders (DSM-5; [71]). This diagnostic shift recognizes the functional limitations pragmatic language impairments can pose on social participation, relationships, and academic/occupational performance.

This study is the first to examine correlates of pragmatic language difficulty in the *FMR1* premutation. Research along these lines may have implications for clinical practice. Although pragmatic deficits of the *FMR1* premutation are generally considered to be subclinical and mild in nature, the presence of these features has been shown to impact outcomes at the family level [14]. Furthermore, pragmatic language difficulties comprise a principal feature of autism and the broad autism phenotype, both of which are elevated in women with the *FMR1* premutation [11]. Clinician knowledge of features that interface with social-communication variation in *FMR1* conditions can be helpful in supporting effective clinical interactions. Successful social-communicative interactions are arguably at the crux of the human condition, as this skill supports an innate human need for social relationships with others [72]. Strong interpersonal skills are associated with a number of life advantages, such as enhanced relationship satisfaction, increased social support, and reduced stress and psychological symptoms [72]. This study sheds light on attention to eye gaze as a powerful social tool that is associated with social-communicative engagement in the general population, and which appears to be aberrant in the *FMR1* premutation. Future research may explore eye gaze processing as a potential marker that may relate to other features of the *FMR1* premutation clinical phenotype, potentially contributing to the identification of vulnerable subgroups.

### Limitations and future directions

This study represents a first attempt to characterize sensitivity to eye gaze in women with the *FMR1* premutation, and number of questions remain open to investigation. First, it is difficult to determine causal relationships with certainty, given the correlational design. In developmental research and theory, the ability to process social gaze is considered a foundation for the later development of social-communication skills, and hence it was assumed that the associations examined here would follow the same direction. However, other directional relationships seem plausible, such as the case where an individual who is aware of their own pragmatic difficulties may learn to compensate by attending more carefully to social signals. We were also unable to sample sensitivity to eye gaze occurring directly during the social-communicative interaction, instead relying on animated simulation of eye contact. While the relationships observed in study controls supports the utility of the stimuli in tapping the intended social-cognitive constructs, the use of more naturalistic stimuli may have yielded different results (e.g., [73]). Future studies might take advantage of new technologies, such as wearable eye-tracking devices, which would allow for the quantification of eye gaze patterns during dynamic social interaction. It should also be noted that the sample was primarily Caucasian and limited to mothers who had a child identified as having fragile X syndrome or the *FMR1* premutation, which may limit generalizability. Finally, there are some limitations associated with relying on non-significant *p* values to establish group equivalency [74, 75]; while the groups in this study did not differ significantly on relevant demographic variables, the use of more rigorous matching criteria would have provided further support of group equivalency.

## Conclusions

In conclusion, this study contributes to the growing characterization of social phenotypes associated with the *FMR1* premutation by documenting altered sensitivity to the eyes in the *FMR1* premutation, characterized by increased salience of averted gaze. Eye gaze is a critical medium through which social information is conveyed, supporting effective communication. Our findings suggest that, unlike study controls, women with the *FMR1* premutation were unable to capitalize on information conveyed through the eyes to enhance social-communicative engagement. This study sheds light on mechanisms associated with pragmatic language variation in adults, and how they may be impaired in specific ways in the *FMR1* premutation. The characterization of altered gaze processing in *FMR1* premutation during earlier developmental periods constitutes an important area of future investigation, given the implications of intervening with this high-risk group during developmentally sensitive periods. Efforts to define phenotypes and mechanisms associated with the *FMR1* premutation have significant public health implications, given the high prevalence of this genetic condition and its relatively undefined clinical profile.

## Abbreviations

*FMR1*: *Fragile X Mental Retardation-1*
FXTAS: Fragile X-associated tremor ataxia syndrome
